# HMGB1 is involved in viral replication and the inflammatory response in coxsackievirus A16-infected 16HBE cells via proteomic analysis and identification

**DOI:** 10.1186/s12985-023-02150-8

**Published:** 2023-08-09

**Authors:** Yajie Hu, Chen Liu, Jinghui Yang, Mingmei Zhong, Baojiang Qian, Juan Chen, Yunhui Zhang, Jie Song

**Affiliations:** 1 Department of Pulmonary and Critical Care Medicine, The First People’s Hospital of Yunnan Province, Kunming, China; 2 The Affiliated Hospital of Kunming University of Science and Technology, Kunming, Yunnan China; 3 Department of Pediatrics, The First People’s Hospital of Yunnan Province, Kunming, China; 4 Institute of Medical Biology, Chinese Academy of Medical Science and Peking Union Medical College, Yunnan Key Laboratory of Vaccine Research and Development on Severe Infectious Diseases, Kunming, China

**Keywords:** Coxsackievirus A16 (CV-A16), Tandem mass tag (TMT)-based quantitative proteomics, Virus‒host interaction, High mobility group box protein 1 (HMGB1), Viral replication, Inflammatory response

## Abstract

Coxsackievirus A16 (CV-A16) is still an important pathogen that causes hand, foot and mouth disease (HFMD) in young children and infants worldwide. Previous studies indicated that CV-A16 infection is usually mild or self-limiting, but it was also found that CV-A16 infection can trigger severe neurological complications and even death. However, there are currently no vaccines or antiviral compounds available to either prevent or treat CV-A16 infection. Therefore, investigation of the virus‒host interaction and identification of host proteins that play a crucial regulatory role in the pathogenesis of CV-A16 infection may provide a novel strategy to develop antiviral drugs. Here, to increase our understanding of the interaction of CV-A16 with the host cell, we analyzed changes in the proteome of 16HBE cells in response to CV-A16 using tandem mass tag (TMT) in combination with LC‒MS/MS. There were 6615 proteins quantified, and 172 proteins showed a significant alteration during CV-A16 infection. These differentially regulated proteins were involved in fundamental biological processes and signaling pathways, including metabolic processes, cytokine‒cytokine receptor interactions, B-cell receptor signaling pathways, and neuroactive ligand‒receptor interactions. Further bioinformatics analysis revealed the characteristics of the protein domains and subcellular localization of these differentially expressed proteins. Then, to validate the proteomics data, 3 randomly selected proteins exhibited consistent changes in protein expression with the TMT results using Western blotting and immunofluorescence methods. Finally, among these differentially regulated proteins, we primarily focused on HMGB1 based on its potential effects on viral replication and virus infection-induced inflammatory responses. It was demonstrated that overexpression of HMGB1 could decrease viral replication and upregulate the release of inflammatory cytokines, but deletion of HMGB1 increased viral replication and downregulated the release of inflammatory cytokines. In conclusion, the results from this study have helped further elucidate the potential molecular pathogenesis of CV-A16 based on numerous protein changes and the functions of HMGB1 Found to be involved in the processes of viral replication and inflammatory response, which may facilitate the development of new antiviral therapies as well as innovative diagnostic methods.

**Supplementary Information:**

The online version contains supplementary material available at 10.1186/s12985-023-02150-8.

## Introduction

Hand-foot-and-mouth disease (HFMD) is a common viral illness caused by a group of enteroviruses, with enterovirus 71 (EV-A71) and coxsackievirus A16 (CV-A16) being the main pathogens in China [1]. Most HFMD patients exhibit a benign, self-limiting illness characterized by skin eruptions on the hands, feet, or buttocks and ulcers or blisters in the mouth with or without fever, but in rare cases, the symptoms can progress into severity, and the sick child can experience serious complications in the central nervous system and even death [2, 3]. Accumulating evidence indicates that EV-A71 is more likely than CV-A16 to lead to severe neurologic and cardiorespiratory problems [4]. Therefore, most of the previous studies focused on EV-A71, whereas CV-A16 was relatively less studied. An inactivated vaccine against EV-A71 was successfully developed, but it does not provide cross protection against other enteroviruses, including CV-A16 [5, 6]. Meanwhile, it was found that the infection rate of CV-A16 has also been high in recent years, but there is no specific drug. Moreover, even though CV-A16 infection usually causes mild symptoms, it can also lead to severe and fatal HFMD cases [7]. Therefore, several vaccine companies and academic institutions have begun to focus on CV-A16 in an attempt to develop monovalent or multivalent CV-A16 vaccines.

In the past decade, transcriptomics has been used to analyze the interactions between viruses and host cells [8]. We also performed an in-depth analysis of the changes in transcriptomics in respiratory epithelial cells following EV-A71 and CV-A16 infections, which also provided useful clues regarding the pathogenesis of HFMD [9]. However, the identification of changes in the transcriptome can only represent the transcriptional level, and the genes that are changed at the transcriptional level cannot directly reflect the situation of their protein levels, while the proteins are what truly works in the body, and the differential protein expression can better reflect the physiological changes of host cells induced by virus infection [10, 11]. In recent years, the tandem mass tag (TMT) labeling-based quantitative proteomic approach has become a popular methodology in explorations of host cellular responses to viral infection, which may provide specific insights into the cellular mechanisms involved in viral pathogenesis [11–13]. For example, TMT-based quantitative proteomics analysis revealed that the dominant nuclear accumulation of viral matrix protein of Newcastle disease virus (NDV) inhibited host cell transcription, RNA processing and modification, protein synthesis, posttranscriptional modification and transport, but the nuclear localization signal mutation in viral matrix protein significantly attenuated the replication ability of NDV by upregulating TIFA/TRAF6/NF-κB-mediated production of cytokines [14]. The proteomic landscape data of Japanese encephalitis virus (JEV)-infected fibroblasts showed that the upregulated proteins were mainly involved in innate immune sensing, interferon responses and inflammation and validated the effects of the DNA sensor cGAS in restricting JEV replication, which not only provided a bird’s-eye view into how fibroblast protein composition is rewired following JEV infection but also demonstrated the critical role of the cGAS-STING axis in mediating an antiviral role against JEV infection [15]. TMT-based quantitative proteomic analysis of ISG15 knockout PK15 cells in pseudorabies virus (PRV) infection has indicated that the differentially expressed proteins were mainly involved in various biological processes and signaling pathways, such as signal transduction, the digestive system, and the PI3K-AKT pathway, and AFP, Vtn, Hsp40, Herc5, and Mccc1 were found to be closely associated with PRV propagation, which may provide new insight into molecular mechanisms for PRV infection and further help us identify potential protein targets for antiviral agents [16]. However, relatively little is known about how CV-A16 affects the host cell proteome and then causes changes in cellular processes. Acutally, researchers are focusing on the development of targeted drugs by exploring key molecules involved in regulating virus‒host interactions, so it is essential to elucidate the molecular pathogenesis underlying virus‒host interactions for the development of new therapeutic strategies [17]. Here, to address this, we utilized TMT-based quantitative proteomics to describe the deregulation of cellular protein expression in 16HBE cells infected with CV-A16 and further analyzed the underlying biological functions of these changed proteins, which may deepen our understanding of the pathogenesis of CV-A16 and further open up new avenues for therapeutic intervention.

## Materials and methods

### Cell lines and viruses

16HBE cells were purchased from the China Center for Type Culture Collection (CCTCC; Wuhan, China) and grown in Dulbecco’s modified Eagle’s medium (DMEM; Invitrogen, USA) supplemented with 10% fetal bovine serum (FBS; Gibco, USA), 1% each of L-glutamine, nonessential amino acids, and sodium pyruvate. Monolayers of 16HBE cells were cultured to 80% confluence in 6-well plates and either mock-incubated or incubated with the CV-A16-G20 strain (subgenotype B, GenBank: JN590244.1), isolated from an HFMD patient in Guangxi, China in 2010, at a multiplicity of infection (MOI) of 0.1 for 2 h at 37 °C in serum-free DMEM. After washing with phosphate buffered saline (PBS, pH 7.4) to remove the unadsorbed virus, the cells were maintained in DMEM with 2% FBS at 37 °C for 24 h. Each group was processed with three independent biological replicates.

### Proteomic sample preparation and quantitative liquid chromatography‒mass spectrometry/mass spectrometry (LC‒MS/MS) analysis

The infected and control cells were collected with cell scrapers and resuspended in lysis buffer (9 M urea, 4% CHAPS, 1% IPG buffer, 1% DTT) containing 1 mM PMSF. Then, 600 μl of the sample was added to 5 mm glass beads (Sigma, USA), agitated with a bead-beater and centrifuged at 12,000 rpm for 15 min at 4 ℃. The supernatant was collected, and the concentrations of protein extracts were determined by the Bradford method with bovine serum albumin (BSA; Sigma, USA) as a standard. The resultant pellets were reduced with 10 mM DTT at 37 °C for 1 h, followed by alkylation with 40 mM iodoacetamide at room temperature in the dark for 30 min. Trypsin was added at a ratio of 1:50 (enzyme:protein, w/w) overnight digestion at 37 °C. The next day, TFA was used to bring the pH down to 6.0 to end the digestion. After centrifugation, the supernatant was subjected to peptide purification using a Sep-Pak C18 desalting column. The digested peptides were desalted by StageTip (Thermo Fisher Scientific, USA) and dried by vacuum centrifugation.

For TMT labeling, the samples were resuspended in 100 μl of 50 mM triethylammonium bicarbonate (TEAB) buffer solution, and 40 μl of each sample was transferred to new tubes. Then, 60 μl of 50 mM TEAB was added for vortex mixing, and 41 μl of anhydrous acetonitrile was added to the TMT reagent (Thermo Fisher Scientific, USA) vial at room temperature. The reagents were dissolved for 5 min and centrifuged. Later, 41 μl of the TMT-labeled reagent (TMT 6-plex) was added to each 100 μl sample for mixing. The tubes were incubated at room temperature for 1 h. Finally, 8 μl of 5% hydroxylamine was added to each sample and incubated for 15 min to terminate the reaction. The labeled peptide solutions were lyophilized and stored at − 80 °C.

TMT-labeled peptide mixtures were fractionated according to the high pH reverse-phase HPLC method with an Agilent Zorbax Extend C18 column (5 µm, 150 mm × 2.1 mm). Hereafter, LC‒MS/MS analysis was conducted using a Triple TOF 5600 System (AB SCIEX, USA). The mobile phases were water with 0.1% formic acid (A) and 99.9% acetonitrile with 0.1% formic acid (B). Chromatographic separation was carried out on a reverse-phase C18 column (3 µm, 15 cm × 75 µm). The delivery flow rate was set at 1 µl/min.

### Proteomic analysis

Proteome Discoverer TM 2.2 (Thermo Fisher, USA) was used for protein identification and quantification using the UniProt human database. Various search parameters were set as follows: trypsin digestion, up to two missed cleavages, a peptide mass tolerance of ± 10 ppm, variable modifications of oxidation (M), a fragment mass tolerance of 0.02 Da. Alkylation on cysteine was considered a fixed modification in the database search. For the protein quantification method, TMT 10-plex was selected. With decoy as the database pattern, a global false discovery rate (FDR) was set to 0.01, and protein groups considered for quantification required Score Sequest HT > 0 and unique peptides ≥ 1. We conducted automatic normalization through PD database search software, and the batch effect was removed by dividing different labeled groups by MIX.

Principal component analysis (PCA) can reduce the complexity of data and dig deeper into the relationship and variation between samples. In the current study, PCA was carried out using all identified proteins to determineliers and distinguish clusters of samples with high similarity. Next, fold change (FC) > 1.5 or FC < 0.67 and Student’s t test *P* value < 0.05 were used as the threshold for screening the proteins with significant differences. FC > 1.5 was defined as upregulated, while FC < 0.67 was defined as downregulated. Additionally, we performed unsupervised hierarchical clustering analysis of these differentially expressed proteins with Multiple Experiment Viewer software. The Euclidean distance measure and the average linkage clustering algorithm were used in this analysis. The branching pattern was illustrated in a dendrogram in which the similarity between the protein expression profiles could be visually assessed.

To further analyze functions and involvement in common biological processes, the final list of nonredundant protein IDs obtained after global quantification was classified into three different categories of Gene Ontology (GO): biological processes (BP), cellular component (CC) and molecular function (MF). The Kyoto Encyclopedia of Genes and Genomes (KEGG) database was used to ascribe identified proteins to particular biological mechanisms and cellular pathways (the established criteria: *P* adjusted < 0.05). GO and KEGG enrichment analyses were performed using the Protein Analysis Through Evolutionary Relationships (PANTHER) Classification System (http://pantherdb.org) and Database for Annotation, Visualization and Integrated Discovery (DAVID) 6.8 (https://david.ncifcrf.gov/home.jsp), respectively. Subsequently, the protein domain structure is a conserved part of a given protein and can function independently of the rest of the protein. To address the domain features of the lysine-acetylated proteins altered by CV-A16 infection, domain annotation and enrichment analysis were performed with InterProScan based on the protein sequence alignment method (http://www.ebi.ac.uk/interpro/). The subcellular localization was predicted using an updated version of PSORT/PSORT II, the WoLF PSORT program (http://www.genscript.com/wolf-psort.html). Finally, protein‒protein interaction (PPI) analysis was performed by GeneMANIA (http://genemania.org/).

### Immunoblot validation of proteomics data

Western blotting (WB) was used to validate the abundance of cellular proteins between CV-A16-treated and untreated 16HBE cells as measured by quantitative proteomic analysis. Briefly, the collected cellular samples were centrifuged at 12,000 rpm for 5 min at 4 °C and washed twice with cold PBS. Then, each sample with approximately 1 × 10^8^ total cells was sonicated three times on ice in RIPA lysis buffer containing a high-intensity ultrasonic processor (Scientz, China) at 270 W for 5 min. The remaining debris was removed by centrifugation at 12,000 rpm at 4 °C for 10 min, and the protein concentration in the cell lysates was measured using a BCA Protein Assay kit (Thermo Scientific, USA). Next, equivalent amounts of protein extracts were subjected to 10 ~ 12% SDS‒PAGE gel and then transferred from the gel to a polyvinylidene difluoride (PVDF) membrane (Millipore, USA) using a Trans-Blot Semi-Dry Transfer Cell (Bio-Rad, USA). The membrane was blocked in 5% skim milk for 1 h to prevent nonspecific binding before incubation with an appropriate primary antibody at 4 °C overnight at dilutions recommended by the manufacturer. The following primary antibodies were used: vitronectin (VTN, 1:1000 dilution; Affinity, USA), ACTN4 (1:2000 dilution; Abcam, USA), filamin-B (FLNB, 1:1500 dilution; Abcam, USA) or glyceraldehyde 3-phosphate dehydrogenase (GAPDH, 1:5000 dilution; Affinity, USA). After washing with Tris-buffered saline with 0.05% Tween-20 (TBST) solution, the membrane was incubated with appropriate dilutions of anti-mouse IgG or anti-rabbit IgG secondary antibodies (Beyotime, China) conjugated to horseradish peroxidase (HRP) in blocking solution for 1 h at room temperature. Following further washes in TBST, immunoreactive bands were visualized by enhanced chemiluminescence using a LumiGLO Chemiluminescent Substrate (Kirkegaard & Perry Laboratories) followed by exposure to X-ray film (Kodak, Japan).

### Immunofluorescence (IF) microscopy

16HBE cells were seeded at 5 × 10^4^ cells per well on a 24-well plate. Where needed, the cells were infected with CV-A16. At 24 h post infection, the slide was then washed with PBS, fixed with 4% paraformaldehyde for 30 min and permeabilized with 0.1% Triton in 5% BSA for 10 min at room temperature. The cells were probed with primary antibodies (VTN, ACTN4, FLNB) diluted at 1:200 in PBS overnight at 4 ℃ followed by incubation with a 1:300 ratio of secondary antibodies for 1 h at room temperature. The cells were washed with PBS twice between each step. Cells were counterstained with DAPI for 5 min at room temperature, and images were visualized by a confocal laser scanning microscope (Leica, Germany).

### Examination of high mobility group box protein 1 (HMGB1)

As a protein that can shuttle between the cytoplasm and nucleus, the expression level and localization of HMGB1 were detected by WB (anti-HMGB1 at 1:1000 dilution; Abcam, USA) and IF (anti-HMGB1 at 1:200 dilution; Abcam, USA) in this study as described above. Meanwhile, the amount of extracellular HMGB1 in the cell culture supernatant was determined using an ELISA kit (Shino-Test Corporation, Japan) according to the manufacturer’s operation manual.

### Transfection and identification of plasmid

To explore the role of HMGB1, overexpression and knockdown plasmids targeting HMGB1 were constructed by GenePharma (Shanghai, China). Meanwhile, the control nontargeted siRNA (NC) was also prepared as the negative control. 16HBE cells were grown to 70% confluency in 6-well plates and then transfected with plasmids using Lipofectamine 2000.

### Detection of the effect of HMGB1 on CV-A16 replication

The effect of HMGB1 on the changes in viral replication dynamics in CV-A16 cells was evaluated by viral load, viral titer, and VP1 expression after transfection with overexpression and knockdown plasmids targeting HMGB1. The viral load and viral titer were evaluated by qRT‒PCR and plaque assays, respectively, as we described previously [18]. VP1 expression was monitored via WB as mentioned above. The primers used in this study were as follows: HMGB1 Forward: 5’-ATGGGCAAAGGAGATCCTAAGAAGC-3’ and Reverse: 5’-TTCATCATCATCATCTTCTTCTTCA-3’; β-actin Forward: 5’-GGGCATGGGTCAGAAGGATT-3’ and Reverse: 5’-TCGATGGGGTACTTCAGGGT-3’.

### Determination of inflammatory cytokines

The supernatants of the cultured cells were harvested for inflammatory cytokine determination with flow cytometry. Then, the levels of 12 inflammatory cytokines, namely, TNF-α, IL-12, IL-4, IL-17, IL-8, IFN-γ, IL-10, IL-1β, IL-6, IL-2, IFN-α and IL-5, were tested with a commercial Bio-Plex cytokine assay (RAISECARE Company, China) in accordance with the manufacturer’s instructions. Finally, the supernatants of different samples were analyzed on a NovoCyte flow cytometer (ACEA Bioscience, USA), and the concentrations of these cytokines were calculated from the corresponding standard in the kit and further analyzed by LEGENDplex v8.0 software.

### Statistical analysis

Data are expressed as the mean ± standard deviation (SD). All results represent three independent experiments. The significance of differences between groups was determined by Student’s t test. Significant differences in all statistical tests were set at 0.05 (*P* < 0.05).

## Results

### Quantitative proteomic analysis of CV-A16-infected 16HBE cells

**Fig. 1:**
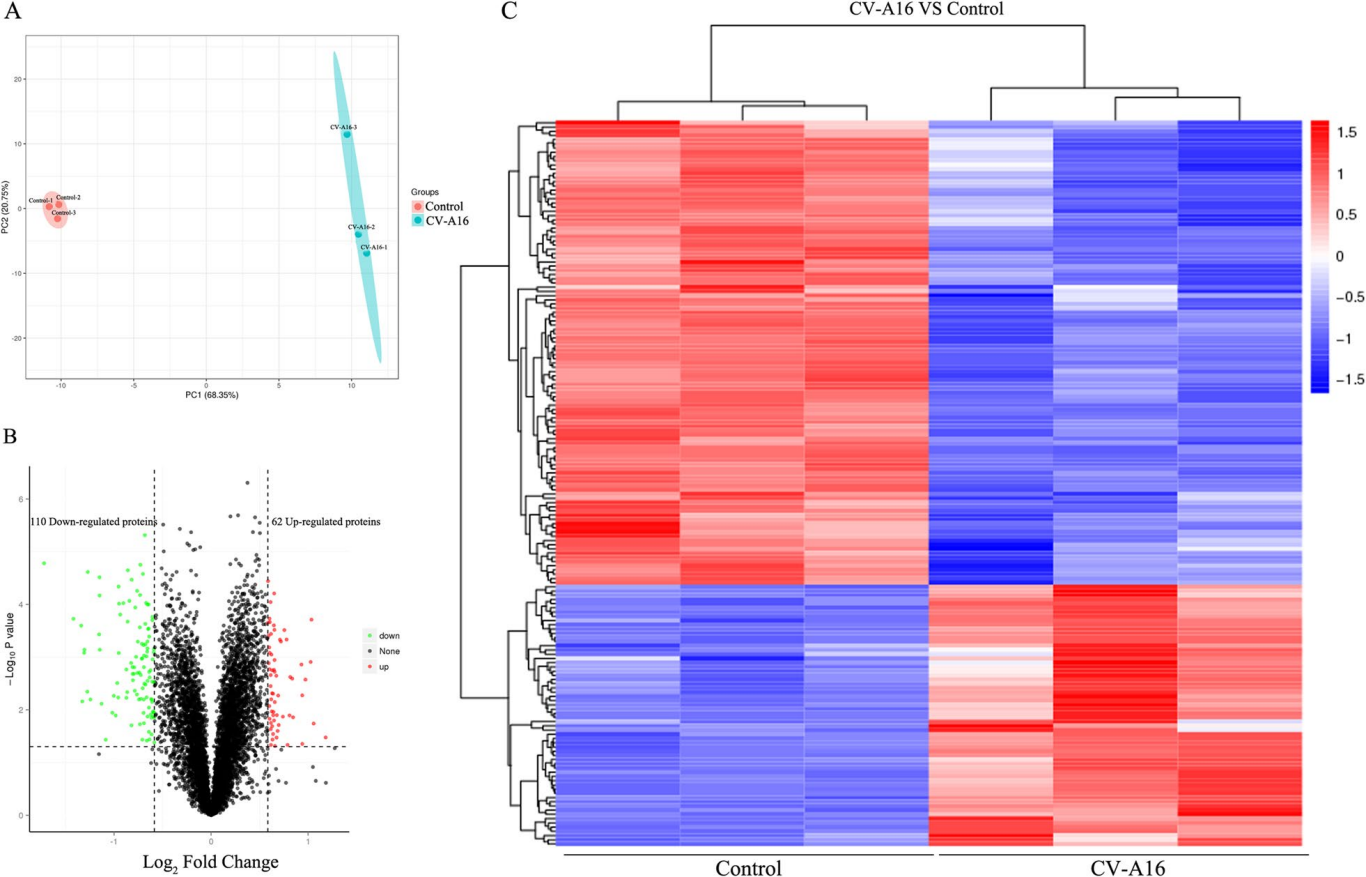
Protein expression patterns of 16HBE cells in response to CV-A16 infection. **A** Principal component analysis (PCA) performed on infected and uninfected samples with three biological replicates. **B** Volcano plot demonstrating the expression of cellular proteins from CV-A16-infected and uninfected cells. Up/downregulated proteins are indicated in red and green, respectively. Proteins not classified as up/downregulated are plotted in black. **C** Heatmap of the significantly differentially expressed proteins. Student’s two-tailed *t* test (P value) was used to compare the means of two groups

The proteomic analysis revealed that there were 6615 proteins in total successfully identified in two groups with three biological replicates each, and the PCA results showed that the CV-A16-infected samples were clearly segregated from the control samples (Fig. 1A). The differentially expressed proteins were screened according to the standard that the expression of multiple changed more than 1.5-fold (upregulated more than 1.5-fold or downregulated less than 0.67-fold) and had a *P* value < 0.05. Among them, 62 differentially expressed proteins were upregulated, and 110 were downregulated (Fig. 1B). The detailed differentially expressed proteins are listed in Additional file 1. Then, these differentially expressed proteins were utilized to perform hierarchical clustering analysis, which illustrated the distinguishable protein expression pattern between the CV-A16- and mock-infected groups (Fig. 1C). Thus, these data suggested that CV-A16 infection can lead to a large number of protein expression changes in host cells, and whether these changes are related to the pathogenesis of CV-A16 is unknown.

### Functional characterization, subcellular localization and network analysis of the differentially regulated proteins

**Fig. 2:**
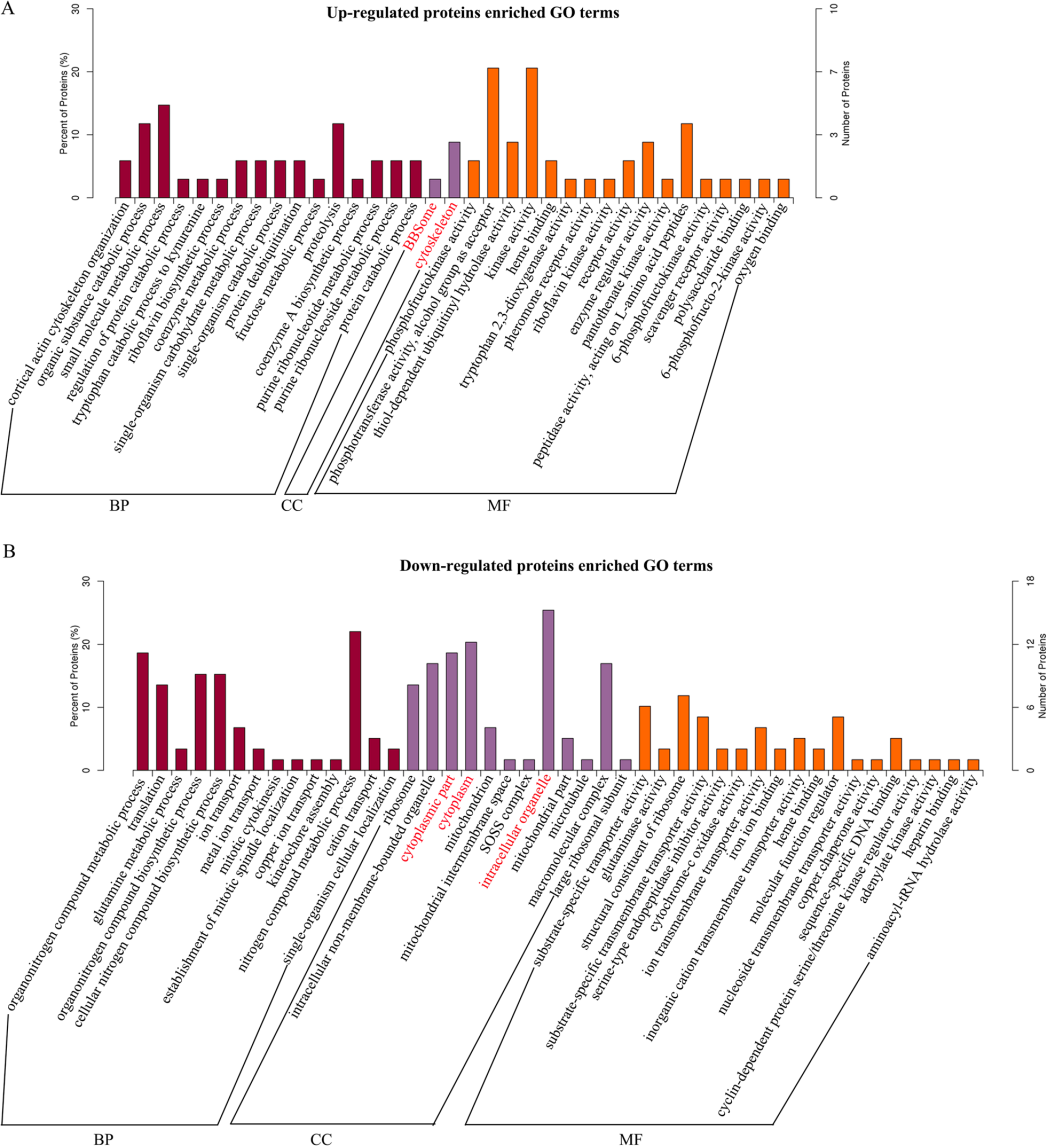
GO functional classification of the differentially expressed proteins. **A** Functional annotations of upregulated proteins. **B** Functional annotations of downregulated proteins

First, to better understand the preferred functional characteristics for the differentially expressed proteins in response to CV-A16 infection, GO analysis based on BP, CC and MF was performed. Upregulated and downregulated differentially expressed proteins were submitted to the PANTHER website separately, with all quantified proteins in this study as background. Categories with *P* values < 0.05 were considered to be over- or underrepresented by regulated proteins. As shown in Fig. 2, 30, 14 and 35 annotations were found in the categories of BP, CC and MF, respectively. In the category of BP, distinct processes of metabolic, catabolic and biosynthetic were mostly enriched. For CC annotation, the upregulated proteins, such as HEL113, were only involved in the BBSome and cytoskeleton, while intracellular organelle, cytoplasm and cytoplasmic part were the top three ranks in the downregulated proteins (Marked in red in Fig. 2). Moreover, it was further observed that the major MF of the dysregulated proteins was related to different activities, such as phosphofructokinase activity, kinase activity, receptor activity, substrate-specific transporter activity, glutaminase activity, and so on. Thus, the functional characteristics of these dysregulated proteins induced by CV-A16 infection might help us to dissect the functional changes in host cells during CV-A16 infection and further provide an underlying mechanism between CV-A16 and the host.

**Fig. 3:**
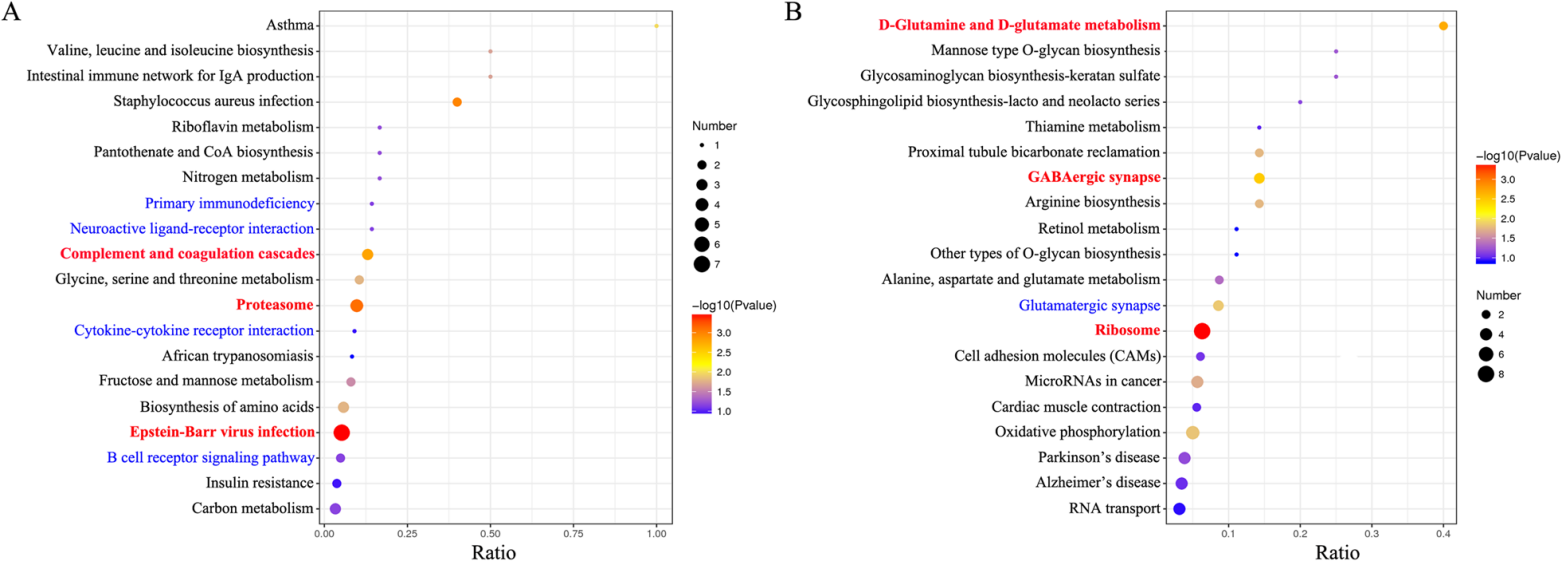
KEGG pathway analysis of differentially expressed proteins isolated from 16HBE cells after CV-A16 infection using the DAVID database. **A** KEGG pathway-based enrichment analysis of upregulated proteins. **B** KEGG pathway-based enrichment analysis of downregulated proteins

Second, to identify the signal transduction pathways affected by CV-A16 infection, KEGG enrichment was carried out. As summarized in Fig. 3, “Epstein‒Barr virus infection”, “proteasome” and “complement and coagulation cascades” were the top 3 enriched pathways among the upregulated KEGG pathways, while “ribosome”, “GABAergic synapse” and “D-glutamine and D-glutamate metabolism” were the top 3 enriched pathways among the downregulated KEGG pathways. Additionally, several pathways associated with immunity were statistically enriched (marked in blue in Fig. 3), such as “primary immunodeficiency”, “cytokine‒cytokine receptor interaction”, and “B-cell receptor signaling pathway”. On the other hand, pathways strongly linked to the central nervous system were also enriched (marked in blue in Fig. 3), such as “neuroactive ligand‒receptor interaction” and “glutamatergic synapse”. Previous studies have demonstrated that CV-A16 infection can induce immune disorders and severe neurological complications [19, 20]. Therefore, these results might provide clues and a theoretical basis for further clarifying the pathogenesis and development of CV-A16 infection.

**Fig. 4:**
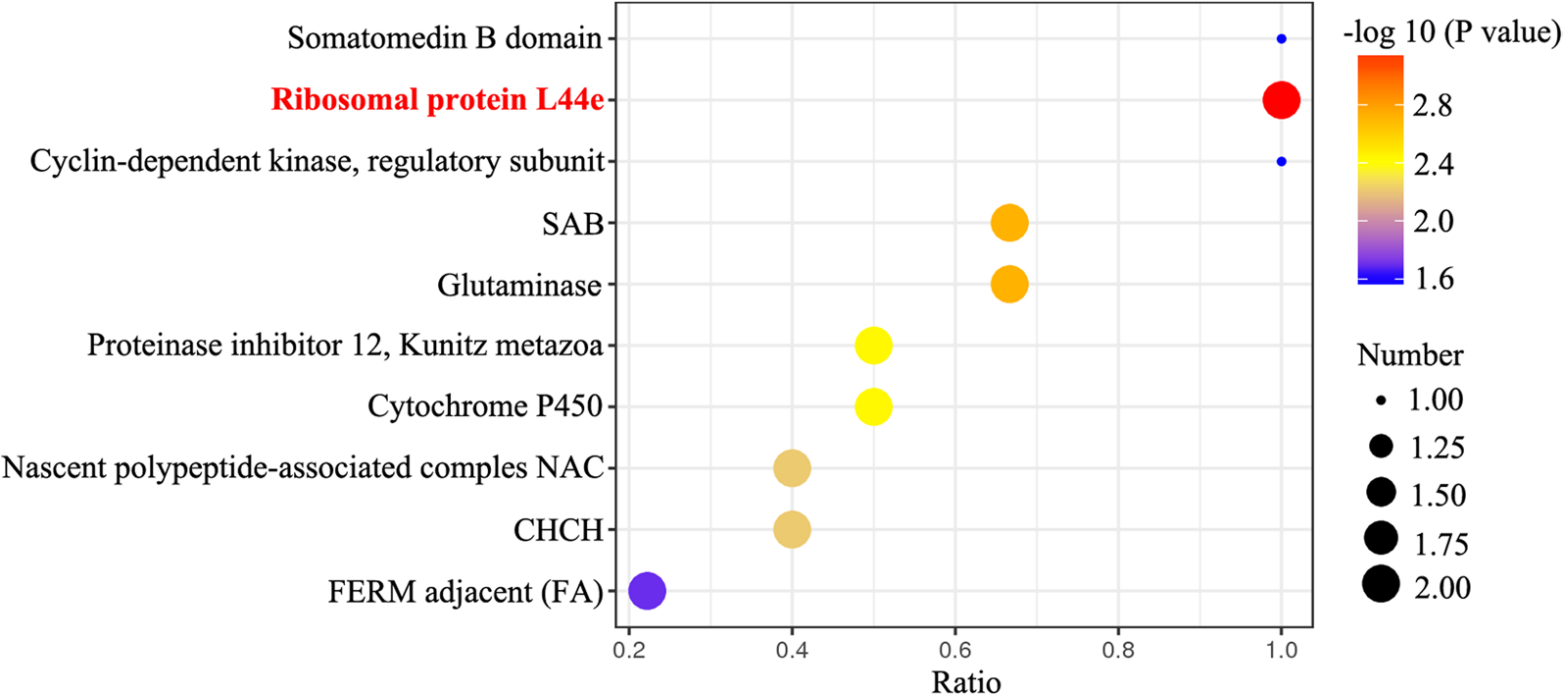
Significantly enriched protein domains of differentially expressed proteins

Third, to find the basic functional units of proteins, the protein domains were analyzed. Under CV-A16 infection, the analysis of significantly enriched domains of the differentially expressed proteins mainly contained 10, namely, the somatomedin B domain, ribosomal protein L44e, cyclin-dependent kinase (regulatory subunit), SAB, glutaminase, proteinase inhibitor I2, Kunitz metazoan, cytochrome P450, nascent polypeptide-associated complex NAC, CHCH, and FERM adjacent (FA) (Fig. 4).

**Fig. 5:**
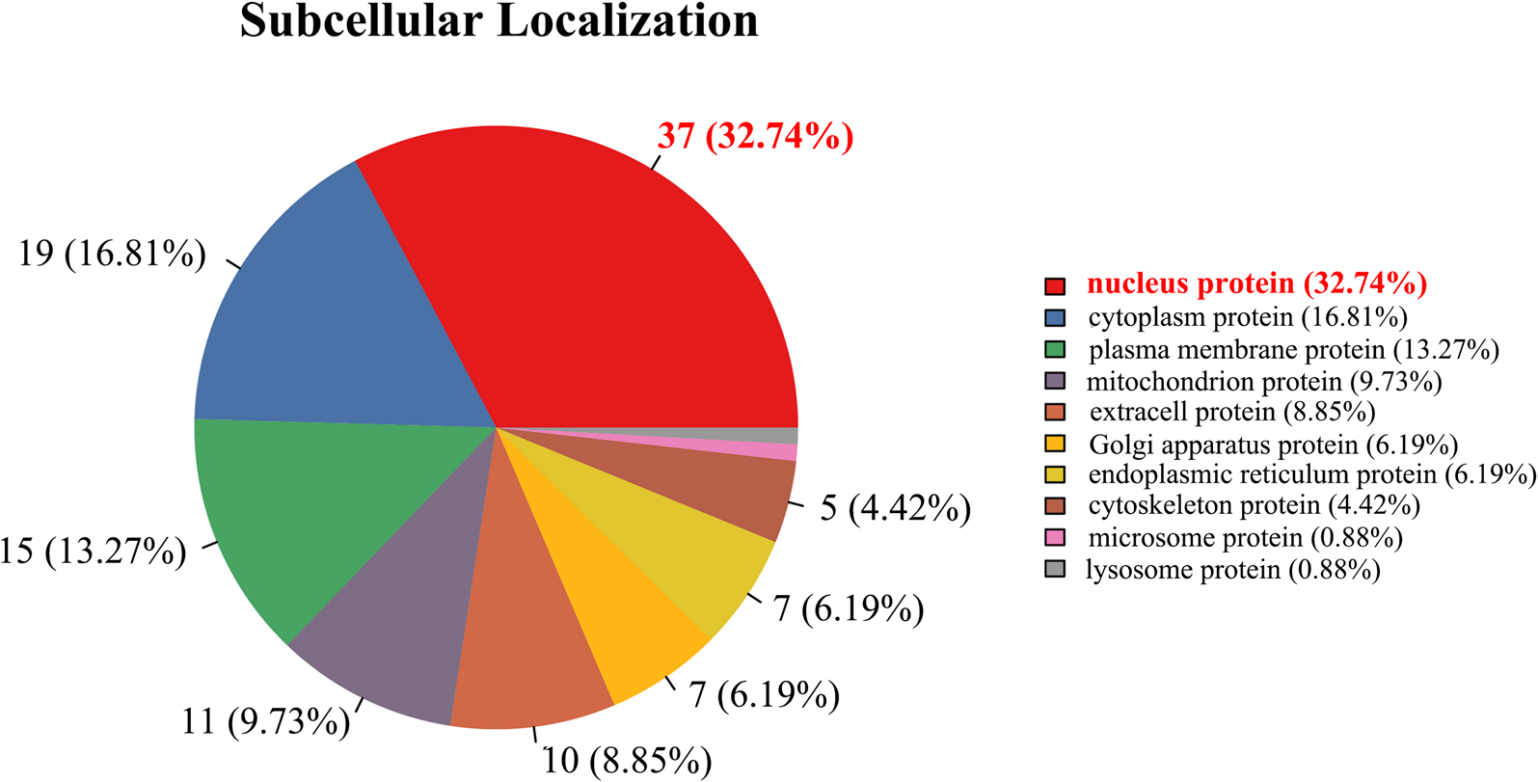
Subcellular localization of differentially modulated proteins was determined based on the PSORT/PSORT II database search

Fourth, to address the cellular distribution properties of these differentially expressed proteins changed by CV-A16 infection, subcellular localization predictions were performed. The results showed that these differentially expressed proteins were located in the nucleus (32.74%), cytoplasm (16.81%), plasma membrane (13.27%), mitochondrion (9.73%), extracell (8.85%), Golgi apparatus (6.19%), endoplasmic reticulum (6.19%), cytoskeleton (4.42%), microsome (0.88%) and lysosome (0.88%) (Fig. 5).

**Fig. 6:**
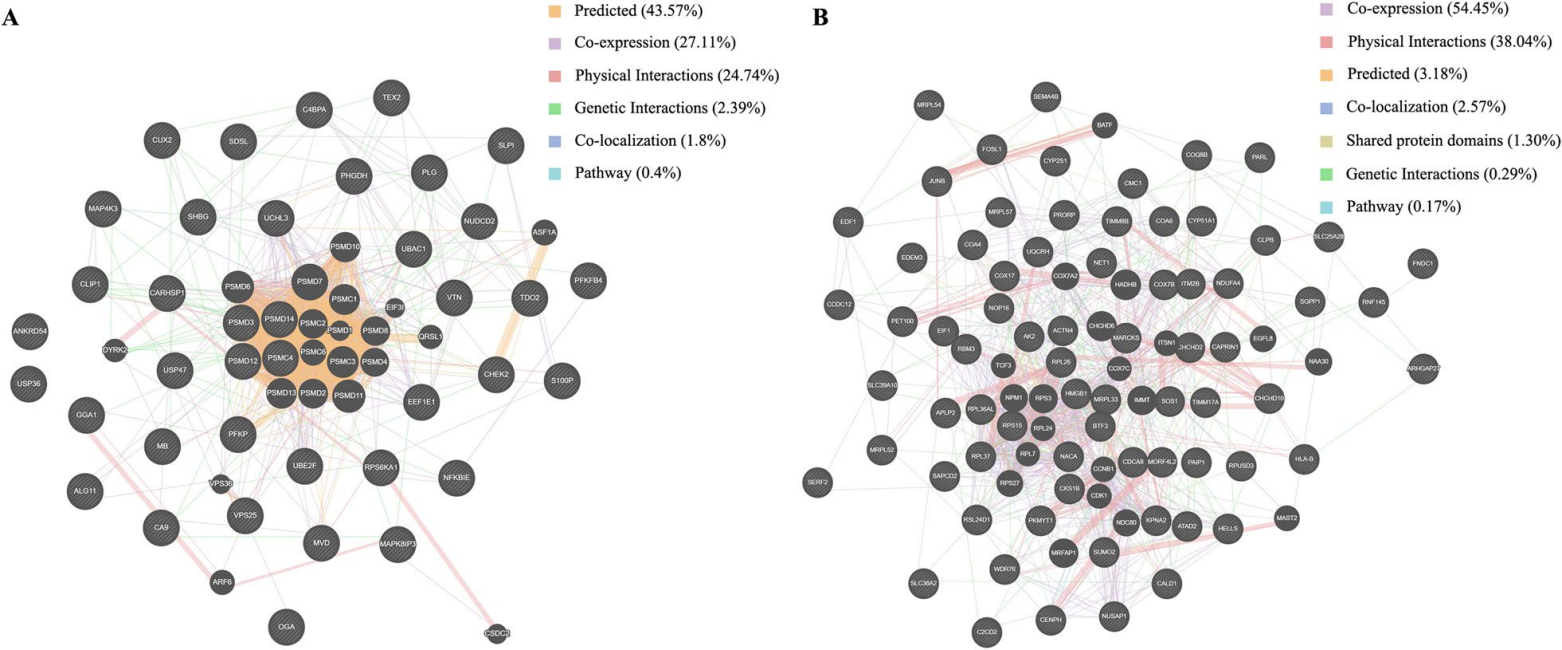
Network interactions of differentially expressed proteins by GeneMANIA. **A** Network of upregulated proteins. **B** Network of downregulated proteins

Finally, to examine the possibility of interactions between differentially expressed proteins and identify important hub proteins among them, network analysis of PPI was conducted. The upregulated proteins were associated with each other through 6 relationships, namely, predicted (43.57%), coexpression (27.11%), physical interactions (24.74%), genetic interactions (2.39%), colocalization (1.8%) and pathway (0.4%), to form a network diagram (Fig. 6A). However, the downregulated proteins were also linked to each other via 7 relationships, namely, coexpression (54.45%), physical interactions (38.04%), predicted (3.18%), colocalization (2.57%), shared protein domains (1.30%), genetic interactions (0.29%) and pathway (0.17%), to build a network diagram (Fig. 6B). Furthermore, the different relationships among these dysregulated proteins are presented with different colors.

### Confirmation of the proteomic data by WB and IF

**Fig. 7:**
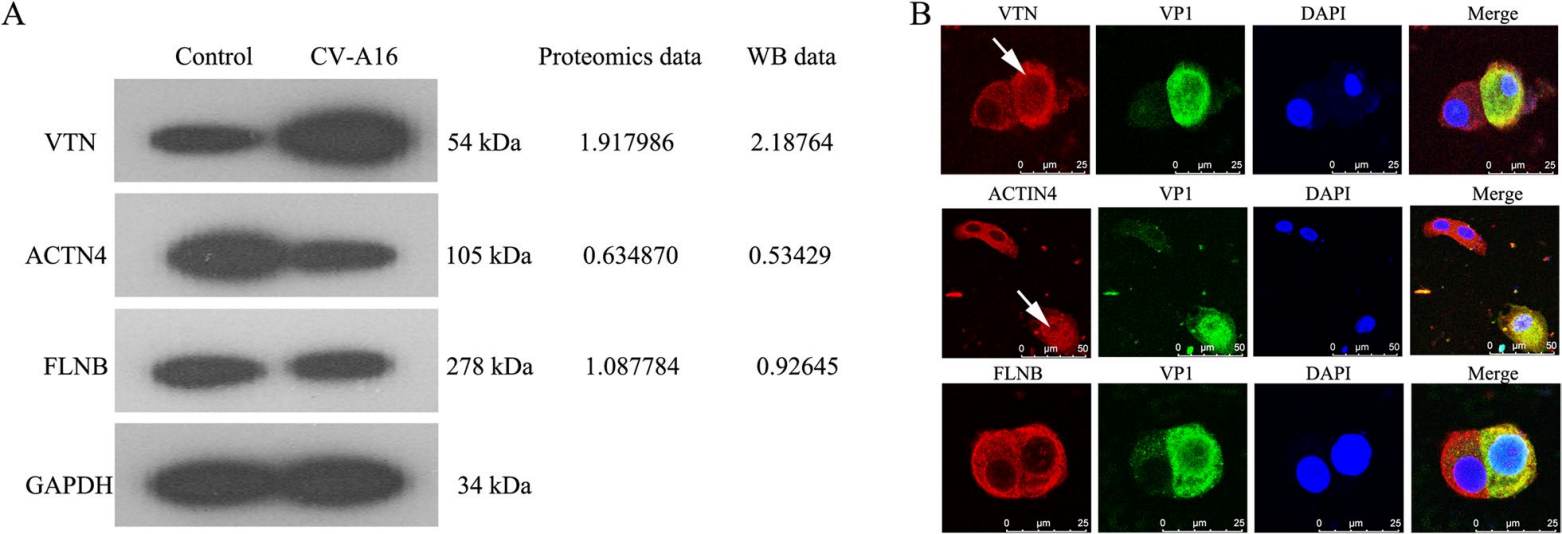
Confirmation of three dysregulated proteins by immunoblot and IF compared to the proteomic analysis. **A** Expression levels of VTN, ACTN4 and FLNB were analyzed by WB. **B** CV-A16-infected 16HBE cells were stained with DAPI to label nuclei (blue), an antibody against CV-A16-VP1 protein to label virus (green) and antibodies against target proteins (red) and examined by confocal microscopy. The white arrows indicate changes in the target protein after infection with CV-A16

To validate the results of the mass spectrometry analysis, three proteins with different relative fold changes and potential functions in CV-A16-induced cellular pathology, namely, VTN, ACTN4, and FLNB, were selected for validation by WB. They represented upregulated, downregulated and unchanged proteins, respectively. As illustrated in Fig. 7A, the WB analysis of these proteins between CV-A16- and mock-infected cells was consistent with the trend in the TMT proteomics data. Moreover, the IF pictures showed that CV-A16 infection enhanced the expression of VTN, reduced the expression of ACTN4 and maintained the expression of FLNB in the cytoplasm (Fig. 7B). Therefore, these results reinforced the reliability of the TMT proteomics data.

### The role of HMGB1 in CV-A16 replication and the inflammatory response

**Fig. 8:**
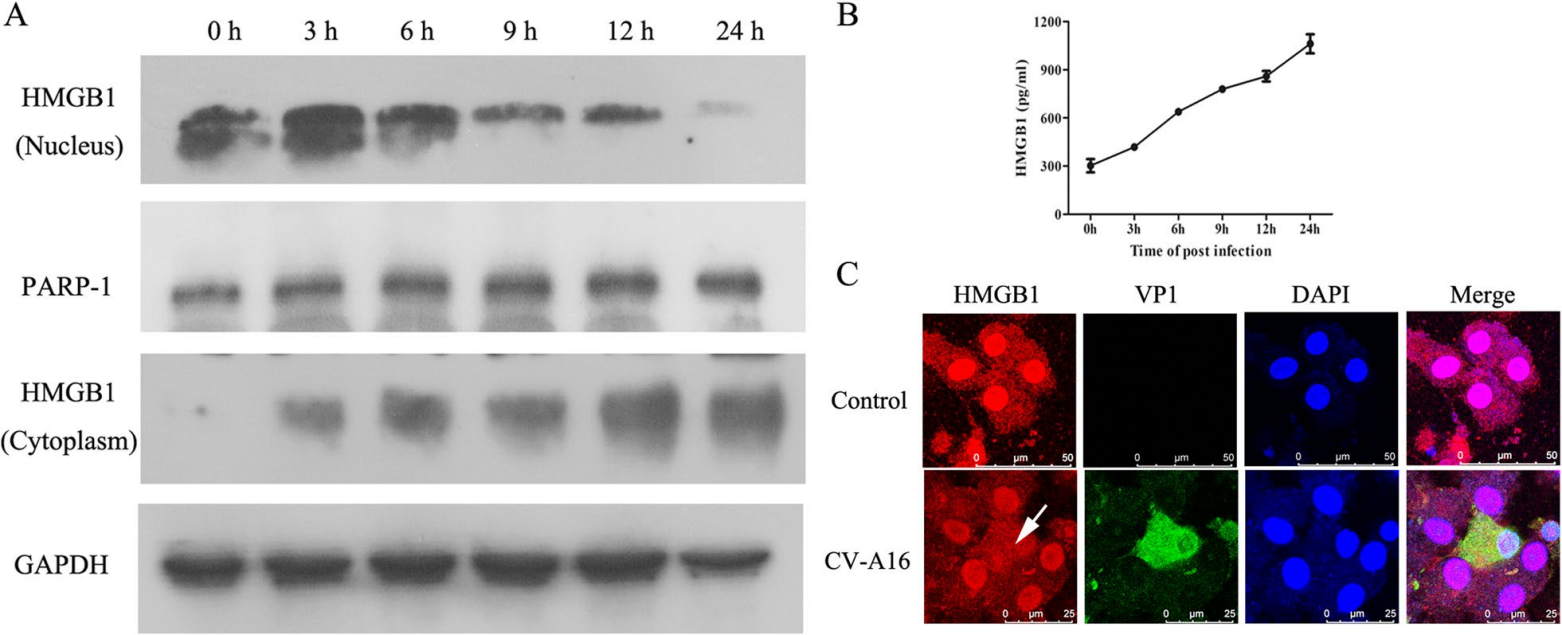
CV-A16 infection induces the release of HMGB1. **A** Nuclear and cytosolic proteins were determined by WB for HMGB1 detection. **B** The extracellular HMGB1 concentration was analyzed via ELISA. **C** The location of HMGB1 was evaluated with IF

HMGB1 is a nuclear DNA-binding protein mainly located in the nucleus, and it can be released into the extracellular space and serve as an important mediator to promote the inflammatory response [21]. In this study, proteome sequencing data showed that CV-A16 infection can lead to the upregulation of HMGB1. Thus, to further explore the influence and significance of HMGB1 during CV-A16 infection, we first examined the expression and localization of HMGB1 in the nucleus and cytoplasm. PARP-1 and GAPDH were considered housekeeping proteins for normalization of data for nuclear and cytosolic HMGB1 expression, respectively. As shown in Fig. 8A, the expression of HMGB1 in the nucleus gradually decreased, but the expression of HMGB1 in the cytoplasm gradually increased. Moreover, the release of HMGB1 was also gradually elevated in a time-dependent manner (Fig. 8B). Meanwhile, the IF images showed that HMGB1, located in the nucleus, was translocated into the cytoplasm under CV-A16 infection (Fig. 8C). Therefore, these results suggest that CV-A16 infection might trigger the release of HMGB1 from the nucleus into the cytoplasm.

**Fig. 9:**
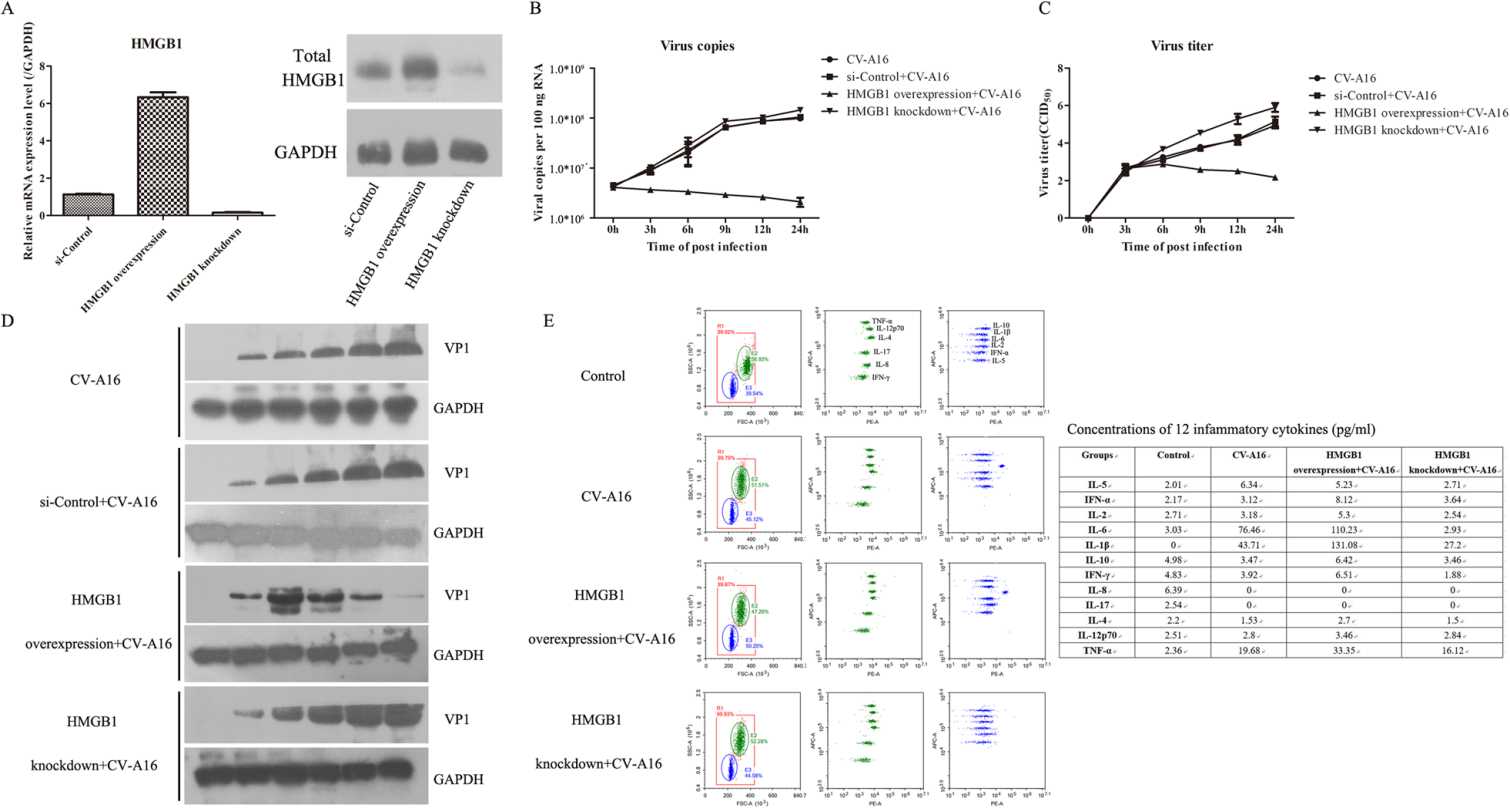
Potential effects of HMGB1 on viral replication and inflammatory responses. **A** HMGB1 mRNA and protein overexpression and knockdown efficiency were detected via qRT‒PCR and WB, respectively. **B** Viral RNA levels were determined by qRT‒PCR. **C** Viral titers were examined by plaque assay. **D** CV-A16 VP1 protein expression was tested with WB. **E** Inflammatory cytokines were measured via flow cytometry

Earlier studies confirmed that many viruses can stimulate the translocation of HMGB1 to restrict viral propagation and further exacerbate the inflammatory response [21]. Therefore, to further explore the role of HMGB1 in virus replication and the inflammatory response, HMGB1 overexpression and knockdown plasmids were constructed and transfected into cells, and the transfection efficiency was also very significant (Fig. 9A). The viral RNA levels and viral titers were determined by qRT‒PCR and plaque assays, respectively. Overexpression of HMGB1 reduced the viral RNA levels and viral titers in CV-A16-infected cells, but inhibition of HMGB1 increased the viral RNA levels and viral titers in CV-A16-infected cells (Fig. 9B, C). Meanwhile, the expression of VP1 displayed a similar trend (Fig. 9D). Ultimately, 12 inflammatory cytokines were detected by flow cytometry. These inflammatory cytokines, especially IL-6, IL-1β and TNF-α, were markedly upregulated in HMGB1-overexpressing cells during CV-A16 infection. In contrast, they were markedly downregulated in HMGB1-knockdown cells during CV-A16 infection. Therefore, these data implied that HMGB1 silencing might result in enhanced viral replication and a weakened inflammatory response.

## Discussion

There are currently no effective antiviral drugs or vaccines available against CV-A16-induced HFMD; therefore, timely and accurate diagnosis and monitoring are crucial for treatment [5]. Previous studies have clearly indicated that in response to viral infection, cells secrete a broad range of proteins through the conventional endoplasmic reticulum-Golgi secretory pathway or nonconventional transport pathways, and these secreted proteins are responsible for the crosstalk among virus-infected and noninfected cells to trigger and boost host cellular processes against viral infection; meanwhile, viruses can also exploit these secreted proteins to facilitate their replication [22, 23]. High-throughput, quantitative proteomic analysis provides a powerful tool for identifying such intricate protein- and pathway-specific alterations in virus-infected cells on a large scale [24, 25]. Thus, in this study, we used TMT-based quantitative proteomic analysis to identify a set of differentially expressed proteins during CV-A16 infection, elucidate the interactions between CV-A16 and host cells, provide new visions and stimulate further investigations on the molecular mechanisms of CV-A16 infection at the proteome level.

Here, we obtained relative quantitative information for 6615 proteins and identified 172 proteins differentially expressed in CV-A16-infected 16HBE cells. Furthermore, our WB and IF results were precisely consistent with those obtained in the TMT-labeled quantitative proteomics analysis of VTN, ACTN4, FLNB in CV-A16-infected 16HBE cells at 24 h. Thereafter, GO enrichment analysis showed that most of the differentially expressed proteins in the BF category were involved in distinct metabolic processes, catabolic processes, and biosynthetic processes. Metabolism is a fundamental biological process consisting of a series of reactions that occur within the cells of living organisms to sustain life and provide energy [26, 27], while catabolic processes are the degradation of complex macromolecules into simpler molecules, and biosynthetic processes are the generation of complex macromolecules, which are jointly responsible for the regulation of cellular homeostasis [28, 29]. Thus, the disturbances in metabolic, catabolic and biosynthetic processes might directly reflect abnormal functions of host cells triggered by CV-A16 infection. “Cytoskeleton” and “intracellular organelle” contained the largest number of differentially expressed proteins in the enrichment CC analysis of upregulated and downregulated proteins, respectively. The cytoskeleton, comprising a network of actin, microtubules, and intermediate filaments, not only provides mechanical support to maintain cell morphology, cell motility, and cell division but also plays an important role in the life cycle of virtually all viruses [30]. For example, cell cytoskeletons have been verified to have close contact with flaviviruses, especially during the process of virus replication [31]. Vaccinia virus was found to take advantage of the actin cytoskeleton to promote viral spread [32]. Herpes simplex virus-1 was also demonstrated to depend on the host cellular cytoskeleton for entry, replication, and exit [33]. Thus, these studies implied that the viruses exploited the host cell cytoskeleton for survival in the host cell and that abnormal cytoskeleton changes would certainly affect host cell-viral interactions. For MF annotation, “phosphotransferase activity, alcohol group as acceptor” and “kinase activity” were most enriched in upregulated proteins, while “structural constituent of ribosome” was mostly enriched in downregulated proteins. Viruses, obligate intracellular parasites, are critically dependent on their hosts to replicate and generate new progeny [34]. Ribosomes exert a direct role in translational regulation in eukaryotes, while the synthesis of viral proteins completely relies on the host translation machinery [35]. Thus, the disruption of ribosomes has a significant effect on the completion of viral protein translation in the host cell. For instance, the translation initiation of hepatitis C virus directly utilizes the translational machinery of host cells [36]. Meanwhile, it was also found that “Ribosome” was also the most enriched KEGG pathway analysis of downregulated proteins. Hence, it was further confirmed that the altered ribosome might play an important role in CV-A16 infection.

Next, KEGG pathway analysis further determined that these differentially expressed proteins were mainly enriched in immune- and neuro-associated pathways, such as primary immunodeficiency, cytokine‒cytokine receptor interaction, B-cell receptor signaling pathway, neuroactive ligand‒receptor interaction, GABAergic synapse, etc. It was reported that inappropriate or excessive activation of the immune reaction plays a crucial role in the regulation of the progression of CV-A16-infected HFMD, including T-cell imbalance and cytokine storm [37, 38]. Moreover, increasing evidence has also demonstrated that CV-A16 is a neurotropic pathogen that has been associated with severe neurological forms of HFMD, such as aseptic meningitis, encephalitis, and acute flaccid paralysis [7]. In fact, previous studies have indicated that infection by neurotropic viruses as well as the resulting immune response can irreversibly disrupt the complex structural and functional architecture of the central nervous system, frequently leaving the patient or affected animal with a poor or fatal prognosis [39]. For example, elevated levels of IL-6 and IL-8 in cerebrospinal fluid might trigger neurological manifestations and poor outcomes in dengue virus infection [40, 41]; Zika virus is a neurotropic virus transmitted via transplacental transmission from mother to fetus, and after entering the fetus, the virus can infect human neural progenitor cells through the AXL protein receptor, leading to changes in the immune pathway in host cells, which further promotes the appearance of neurological symptoms [42]. Hence, the changes in immune- and neuro-associated pathways might be the underlying molecular mechanisms of CV-A16 infection.

Afterward, the protein domains and subcellular localization of these differentially expressed proteins were further analyzed. Protein domains are the basic functional units of proteins, and in recent years, an increasing number of studies have begun to study the interaction network between protein domains to identify potential proteins related to diseases [43]. Our results showed that among the top 10 protein domains, the protein domain with the greatest significance and the largest number of proteins was ribosomal protein L44e. It has been reported that the ribosomal protein L44e family plays a protein cross-linking role in all eukaryotes [44], which is the basis of the interaction between proteins; therefore, the altered ribosomal protein L44e might directly affect the protein interaction in host cells during CV-A16 infection. In addition, assigning the subcellular location of a protein is of paramount importance in the elucidation of its role and in the refinement of knowledge of cellular processes by tracing certain activities to specific organelles [45]. There were 8 subcellular localizations of these dysregulated proteins, and the dysregulated proteins in infected cells were mainly located in the nuclei. It is well known that changes in protein localization have much to do with their functions and the pathways in which they are actively involved [46]. For instance, in its inactive form, NF-κB is sequestered in the cytoplasm, while in its active form, the exposure of the nuclear localization signals on NF-κB subunits and the subsequent translocation of the molecule to the nucleus further activate its downstream factors [47]. Therefore, changes in the subcellular localization of these differentially expressed proteins induced by CV-A16 infection might mean that they trigger changes in their corresponding functions. Finally, to gain further insights into the relationship of virus and host cells, the dysregulated proteins were used to establish a network. These two complex network diagrams indicated that the abnormally expressed proteins were closely related to each other, and these intricate relationships might be the key reasons leading to the pathogenesis of CV-A16. It has also been verified that the progression of viral diseases is not only caused by the differential expression of one protein but is often the result of the interaction of multiple proteins. The detection of proteomics may directly reflect the changes in a whole multifactor and provide a better reference for us to determine the potential pathogenesis.

As we described above, the alteration of nuclear proteins plays an important role in all dysregulated proteins. However, the proteins in the nucleus are not completely located in the nucleus, and some proteins can shuttle between the nucleus and the cytoplasm to regulate overall cell activity [48]. High mobility group protein 1 (HMGB1), a highly conserved DNA-binding protein, is widely expressed in the nuclei of various tissue cells and is involved in the construction and stabilization of nucleosomes and gene transcription in the nucleus [49]. In addition to functions in the nucleus, HMGB1 plays a significant role in inflammation, immunity, cell growth and death in the extracellular milieu [49]. Thus, HMGB1 can shuttle between the nucleus and cytoplasm in different situations to perform different biological functions. Previous studies have mainly focused on the function of HMGB1 as a nuclear protein. In 1999, HMGB1 was first reported to participate in the pathogenesis of sepsis as a new potential late-stage inflammatory mediator [50], which led researchers to recognize the significance of HMGB1 as an inflammatory factor. Many previous studies have discovered that many viruses do not directly cause cytopathic changes but can cause host cells to release inflammatory cytokines and trigger an inflammatory response, which might induce many viral diseases to form inflammatory cytokine storms and even one of the main causes of death [51]. When the amount of inflammatory factors is moderate, it can resist virus invasion, but the production of excessive inflammatory factors will cause serious pathological damage [52]. In recent years, the inflammatory effect of HMGB1 has received increasing attention in viral diseases. For example, NDV infection triggers HMGB1 release to promote the inflammatory response, which largely contributes to pathogenic damage [53]. Dengue virus exploits HMGB1-mediated autophagy to enhance virus propagation [54]. JEV activates the MAPK pathway by restricting HMGB1 expression for viral replication [55]. Hence, HMGB1 is expected to be a promising therapeutic target for viral diseases. Moreover, HMGB1 was also demonstrated to exert a certain role in other enterovirus infections. For instance, the serum HMGB1 level was significantly increased in EV-A71-induced HFMD, and it was closely associated with severe and critical HMFD patients, but there was no significant change between normal and mild HMFD patients, which suggested that HMGB1 might be involved in the inflammatory pathogenesis of EV-A71-induced severe and critical HFMD patients and that the serum level of HMGB1 could be applied as a clinical indicator for the severity of HFMD [56] Moreover, our previous study also demonstrated that HMGB1 might be a key host factor involved in CV-A10 replication (unpublished data). However, the influence of HMGB1 on CV-A16 infection is unclear. In our current study, we also found that HMGB1 was altered after CV-A16 infection from the proteome data. Therefore, we also focused our attention on HMGB1 in the present study. It should be noted that the key reason why HMGB1 acts as a cytokine and then causes inflammatory responses during virus infection is that HMGB1 is released from the nucleus to the extracellular milieu [57]. In this work, we preliminarily investigated the role of HMGB1 in viral replication and inflammatory formation of CV-A16. Our results showed that HMGB1 was decreased after CV-A16 infection, while overexpression of HMGB1 suppressed CV-A16 replication and promoted the inflammatory response, and vice versa. Therefore, these findings definitely stated that HMGB1 exerted a proinflammatory effect in CV-A16-induced inflammatory pathological injury and a negative regulation of CV-A16 replication.

## Conclusion

In summary, investigating differentially expressed cellular proteins resulting from viral infection is an important means for better understanding virus‒host interactions. In this study, we are the first to report a comprehensive view of host proteomic changes in CV-A16-infected 16HBE cells using TMT-based methodology. We also showed that CV-A16 infection induced significant changes in 172 proteins in 16HBE cells. In addition, bioinformatics analysis of these differentially expressed proteins revealed some of their possible effects on biological functions, pathways, protein domains, subcellular locations, and PPI networks, which might provide new clues to better understand the relationship between virus and host after CV-A16 infection and further explore the pathogenesis of CV-A16. Finally, from the different proteins, we selected HMGB1 for further study and verified that HMGB1 is involved in viral replication and the inflammatory response during CV-A16 infection. Therefore, our high-throughput, unbiased quantitative proteomics study reinforces our understanding of the molecular basis of the CV-A16-host interaction, which may help identify therapeutic targets for CV-A16 infection.

### Supplementary Information

**Additional file 1**. List of differentially expressed proteins

## Data Availability

All data generated or analyzed during this study are included in this published article.

## Abbreviations

HFMD: Hand, foot and mouth disease
EV-A71: Enterovirus 71
CV-A16: Coxsackievirus A16
TMT: Tandem mass tag
JEV: Japanese encephalitis virus
PRV: Pseudorabies virus
LC–MS/MS: Liquid chromatography–mass spectrometry/mass spectrometry
TEAB: Triethylammonium bicarbonate
PCA: Principal component analysis
FC: Fold change
GO: Gene ontology
BP: Biological processes
CC: Cellular component
MF: Molecular function
KEGG: Kyoto Encyclopedia of Genes and Genomes
PANTHER: Protein Analysis Through Evolutionary Relationships
DAVID: Database for Annotation, Visualization and Integrated Discovery
PPI: Protein–protein interaction
WB: Western blotting
SD: Standard deviation
